# Manufacturing and preclinical validation of CAR T cells targeting ICAM-1 for advanced thyroid cancer therapy

**DOI:** 10.1038/s41598-019-46938-7

**Published:** 2019-07-23

**Authors:** Yogindra Vedvyas, Jaclyn E. McCloskey, Yanping Yang, Irene M. Min, Thomas J. Fahey, Rasa Zarnegar, Yen-Michael S. Hsu, Jing-Mei Hsu, Koen Van Besien, Ian Gaudet, Ping Law, Nak Joon Kim, Eric von Hofe, Moonsoo M. Jin

**Affiliations:** 1 Molecular Imaging Innovations Institute, Department of Radiology, New York, NY 10065 USA; 2 Department of Surgery, New York, NY 10065 USA; 3 Department of Pathology and Laboratory Medicine, New York, NY 10065 USA; 4 Department of Hematology/Oncology, Weill Cornell Medicine, New York, NY 10065 USA; 5 Miltenyi Biotec Inc., Sunnyvale, CA 94089 USA; 6 AffyImmune Therapeutics, Inc., Natick, MA 01760 USA

**Keywords:** Immunotherapy, Cancer immunotherapy

## Abstract

While the majority of thyroid cancer patients are easily treatable, those with anaplastic or poorly differentiated recurrent thyroid carcinomas have a very poor prognosis with a median survival of less than a year. Previously, we have shown a significant correlation between ICAM-1 overexpression and malignancy in thyroid cancer, and have pioneered the use of ICAM-1 targeted CAR T cells as a novel treatment modality. For clinical translation of this novel modality, we designed CAR T cells possessing micromolar rather than nanomolar affinity to ICAM-1 to avoid cytotoxicity in normal cells with basal levels of ICAM-1 expression. Herein, we report the automated process of CAR T cell manufacturing with CliniMACS Prodigy (Miltenyi Biotec) using cryopreserved peripheral blood leukocytes from apheresis collections. Using Prodigy, thawed leukopak cells were enriched for CD4^+^ and CD8^+^ T cells, subjected to double transduction using lentiviral vector, and expanded in culture for a total of 10 days with a final yield of 2–4 × 10^9^ cells. The resulting CAR T cells were formulated for cryopreservation to be used directly for infusion into patients after thawing with no further processing. We examined cross-reactivity of CAR T cells toward both human and murine ICAM-1 and ICAM-1 expression in human and mouse tissues to demonstrate that both efficacy and on-target, off-tumor toxicity can be studied in our preclinical model. Selective anti-tumor activity in the absence of toxicity provides proof-of-concept that micromolar affinity tuned CAR T cells can be used to target tumors expressing high levels of antigen while avoiding normal tissues expressing basal levels of the same antigen. These studies support the initiation of a phase I study to evaluate the safety and potential efficacy of micromolar affinity tuned CAR T cells against newly diagnosed anaplastic and refractory or recurrent thyroid cancers.

## Introduction

Adoptive cellular therapy using chimeric antigen receptor (CAR) T cells has benefited from significant technological advancements and lead to impressive clinical outcomes in selected hematological malignancies^1–3^. Due to the scarcity of targetable antigens in the solid tumor setting and concerns of off-tumor reactions of immune cells with healthy tissues, the successful application of CAR T cells to solid cancers has been limited^4^. Recently, we reported a significant correlation between intercellular adhesion molecule (ICAM)-1 expression and the malignant features of thyroid cancer^5^, including BRAF point mutations (V600E)^6^. Although, most thyroid cancers are indolent and curable with standard treatments, 5 to 10% of patients develop progressive disease that are metastatic and refractory to current therapies^7^.

As a new treatment modality for advanced thyroid cancers, we demonstrated a single infusion of CAR T cells specific to ICAM-1 to be highly efficient in eliminating thyroid tumors in preclinical models, providing long lasting survival benefit^5^. Previously, we constructed CAR T cells possessing 10^6^-fold difference in affinity to ICAM-1 (equilibrium constant, *K*_*D*_) from millimolar to nanomolar to find an optimal range of CAR affinity for CAR T killing to be selective to ICAM-1 high tumors^8^. This study had shown that CAR T cells with micromolar affinity rather than more typical low nanomolar affinity to antigens were able to safely eliminate anaplastic thyroid cancer cells while avoiding healthy tissues. Due to cross-reactivity of our CAR T cells between human and mouse ICAM-1, the lack of on-target, off-tumor toxicity by micromolar affinity CAR T cells in mice with human tumor xenografts could be used to support specificity of our CAR T cells toward ICAM-1 high tumors in humans.

In an effort to enhance the safety profile and to better understand the cause for different treatment responses of solid cancers to CAR T cells, we designed a CAR construct that co-expresses the somatostatin receptor type 2 (SSTR2) in T cells to permit imaging of CAR T cell distribution in patients using the FDA-approved PET radiotracer ^68^Ga-DOTATATE^9^. Our previous study also highlighted the value of *in vivo* CAR T cell imaging for both efficacy and safety monitoring by demonstrating CAR T cell expansion concurrent with the onset of tumor reduction and subsequent contraction of T cell numbers once the tumors had been eliminated^8,10^.

For the clinical translation of this novel modality, herein we report development of an automated process of CAR T cell manufacturing using CliniMACS Prodigy^11^. Cryopreserved leukapheresis cells (leukopak) were used as the starting material, which underwent user-defined steps of cell wash to remove cryoprotectant, CD4^+^/CD8^+^ T cell enrichment, T cell activation, transduction by lentiviral vectors, media exchange, culture expansion, and final harvest. Cell samples were obtained prior to transduction and during cell expansion post transduction to assess cell growth, viability, vector copy number (VCN), percent transduction, and T cell surface markers. The final cell products were then formulated for cryopreservation. To approximate the clinical use of CAR T cells in patients, cryopreserved CAR T cells were then thawed and used immediately for intravenous infusion into mice bearing tumor xenografts to evaluate efficacy and safety.

## Results

### Design of CAR T vector

**Figure 1:**
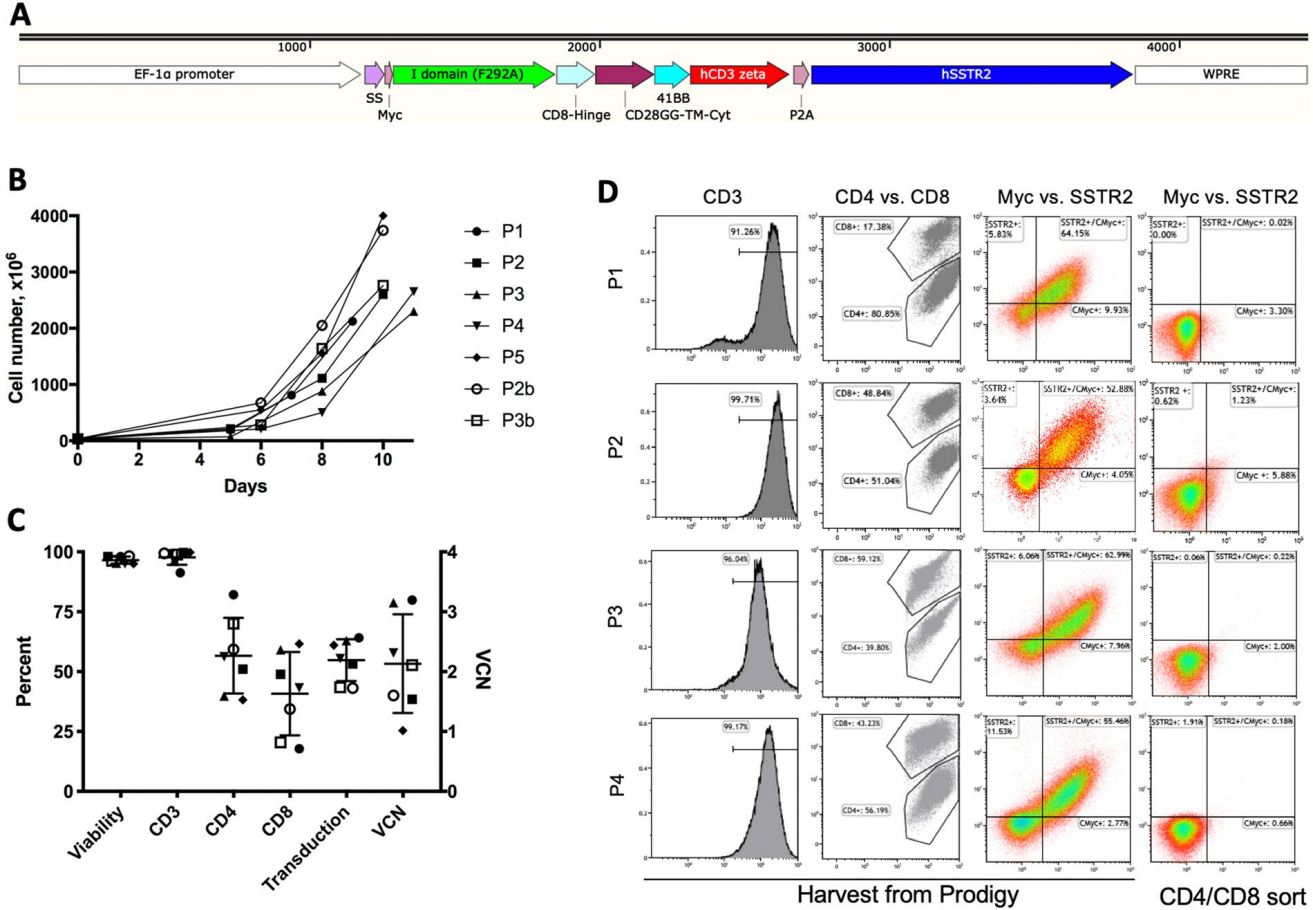
Comparison of Prodigy manufactured CAR T cells for cell expansion, viability, transduction, VCN, and subsets. (**A**) A schematic of the insert sequence of AIC100 lentiviral vector. SS = signal sequence; TM = transmembrane; Cyt = cytosolic domain; hSSTR2 = human SSTR2. DNA ruler is shown above features. (**B**,**C**) Cell expansion and viability were quantified manually by hemocytometer. CD3 and CD4/CD8 subsets, and CAR positive cells were analyzed by flow cytometer. VCN was determined by ddPCR. (**D**) CAR expression was determined by dual labeling of cells by anti-Myc and anti-SSTR2 antibodies for CD4/CD8 sorted cells (day 0) and T cells harvested from Prodigy (day 9, 10, or 11).

The lentiviral vector specific to ICAM-1 is composed of the Inserted (I) domain variant (Gly128-Gly311 containing a point mutation of F292A to bind ICAM-1 at 20 μM) of integrin α_L_β_2_ (also known as lymphocyte functional associated (LFA)-1), the CD8 hinge and transmembrane domain, the intracellular domains of costimulatory CD28 (with mutated dileucine motif^12,13^), 4-1BB, and CD3ζ (Fig. 1A). To image CAR T cells in the body, SSTR2 is concurrently expressed with the CAR via the ribosome skipping sequence P2A^14^. The order of CAR and SSTR2 is different from the design used in our previous study^8^: in the current construct (AIC100), SSTR2 is placed after the CAR with P2A in the middle (Fig. 1A). Myc tag is fused to CAR at the N-terminal to allow for detection of CAR expression by anti-Myc antibody. The titer of the virus was determined by the manufacturer (Lentigen) to be 4.3 × 10^8^ transduction units (TU)/ml.

### AIC100 manufacturing

We have chosen to use the CliniMACS Prodigy system for a closed, automated and robust CAR T cell manufacturing process^11^. The process for Prodigy began with thawing the 60 ml of cryopreserved leukopak and diluting it with 120 ml of PlasmaLyte A/1% human serum albumin (HSA) and reducing the DMSO content to 2.2% (v/v). Diluted leukopak was then welded to an application bag, washed to remove DMSO, and sorted to enrich T cells using CD4 and CD8 CliniMACS reagents. ~30 × 10^6^ sorted cells in 70 ml were used to start the process of CAR T manufacturing. T cell transduction with lentiviral vectors was performed twice, 24 and 48 h after the addition of T cell activation reagent, TransAct. Out of a total of 7 runs (designated as P1, P2, P2b, P3, P3b, P4, P5; numeric refers to leukopak of unique donors), 2 runs (P2b, P3b) were performed with spinoculation. CAR T cells were harvested in PlasmaLyte A/1% HSA solution after 9–11 days of culture in Prodigy and cryopreserved after dilution in 2X volume of Cryostor CS10 at a final density of 30–150 × 10^6^ T cells/ml.

### *In vitro* activity of AIC100

Prodigy manufactured CAR T cells were subjected to various qualification and functional assays (n = 7): cell viability, cell number, CAR expression, VCN, T cell subset and phenotype, and effector to target (E to T) assays. Consistent with reported values of cell viability and production yield of CAR T manufacturing using Prodigy^11^, our manufacturing protocol produced a 55 ± 9% transduction rate, 96.5 ± 1.5% viability, 2.9 ± 0.7 × 10^9^ final cell number, corresponding to ~100-fold expansion over ~10-day manufacturing (Fig. 1B–D). The majority of cells in the final product were CD3 positive (98 ± 3.0%) with CD4^+^ and CD8^+^ T cells accounting for 57 ± 16% and 41 ± 17%, respectively. The VCN was in the range of 1.0–3.2 (2.1 ± 0.8), well below the preset criteria of maximum 5 copies per cell.

**Figure 2:**
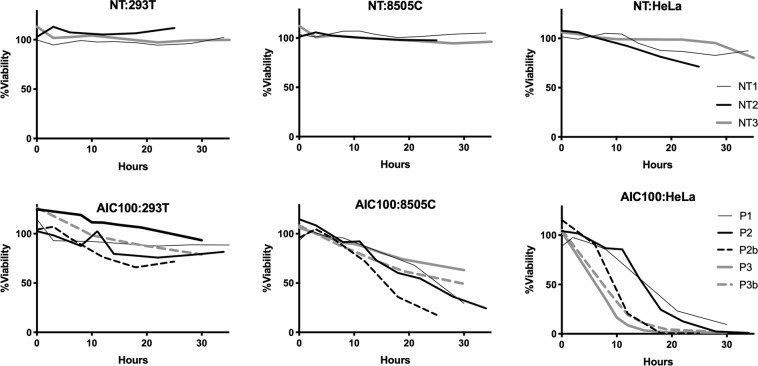
E to T assays of NT and AIC100 cells against ICAM-1 293T (ICAM-1 negative), ATC 8505C, and HeLa cells. The ratio of T cells to target cells was 2.5:1. Viability of target cells treated with different T cells was normalized to target-only cells at corresponding time points. Cell viability was quantified by luminescence readouts.

Different batches of AIC100 after a freeze-and-thaw cycle were then tested for cytotoxicity against 293T (ICAM-1 negative control), 8505C (anaplastic thyroid cancer (ATC); ~10^5^ molecules per cell) and HeLa (cervical cancer cell; ~10^6^ molecules per cell)^8^. AIC100 cytotoxicity was specific with no significant killing of ICAM-1 negative 293T cells and faster killing of HeLa than 8505C (Fig. 2). Specificity and killing rates of AIC100 were similar to previously reported F292A CAR T cells that were manufactured in standard tissue culture plates^8^. As anticipated, non-transduced T cells (NT) produced minimal killing of target cells.

### *In vivo* activity of AIC100

**Figure 3:**
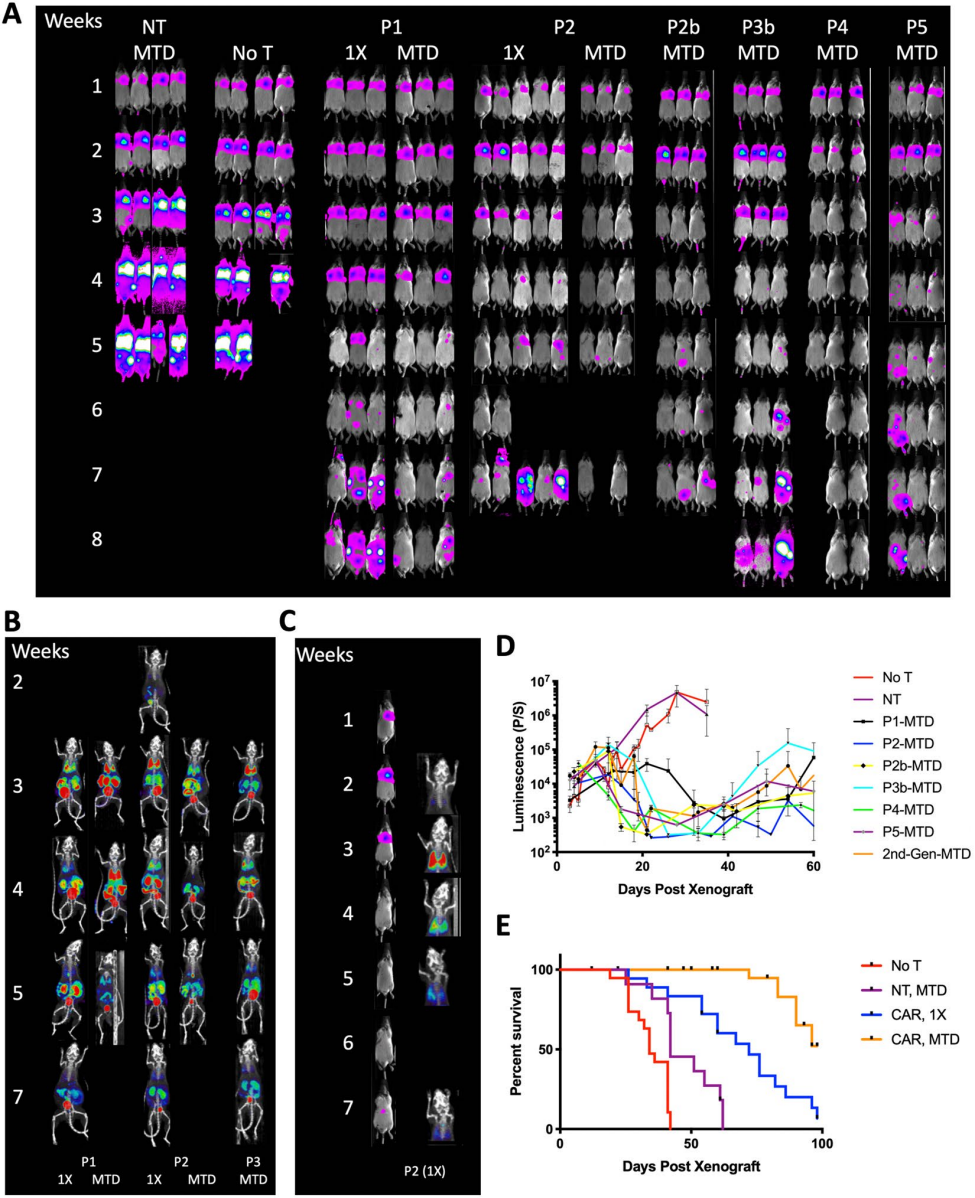
Assessment of *in vivo* activity of AIC100 by tumor elimination, T cell imaging, and survival. (**A**) A weekly measurement of whole body luminescence to detect tumor growth or killing by AIC100. Luminescence intensity is pseudocolored in the range of 10^3^–10^5^ photons/sec/mm^2^. Time elapsed after tumor xenograft is shown in weeks. (**B**) PET/CT imaging by ^18^F-NOTAOCT to image CAR T cell distribution. Images are maximum intensity projections of the entire mouse body (~20-mm-thick plane). PET intensity is pseudocolored in the range of 1–10% injection dose/cm^3^. (**C**) Longitudinal imaging of tumor and AIC100 distribution dynamics, illustrating the occurrence of T cell peak (week 3) post tumor peak (week 2), followed by contraction of T cells. (**D**) Tumor burden in lungs was estimated by the level of luminescence. Mice were treated with AIC100 (P1 to P5 batches used at 1X or MTD), no T cells (No T), non-transduced T cells at MTD (NT), or F292A CAR 2^nd^ generation (41BB-CD3ζ) at MTD. (**E**) Survival curves of 8505C xenografted mice comparing no treatment (n = 19), treatment with NT T cells (n = 11), and treatment with 1X (n = 19) and MTD (n = 27) CAR T cells until 100 days post xenograft.

The efficacy and toxicity of AIC100 were evaluated in non-obese diabetic/Cg-*Prkdc*^*scid*^ *Il2rg*^*tm1Wjl*^/SzJ (NSG) mice (Jackson Laboratory) with xenograft of 0.5 × 10^6^ 8505C ATC cells. At 5 days post xenograft, mice were treated with a single injection of AIC100 (1X dose = 1 × 10^6^ live CAR T; maximum tolerable dose (MTD) = ~10^7^ live T cells = ~5 × 10^6^ live CAR T), NT as a control at MTD, or left untreated (Fig. 3). MTD was defined as a dose of cells that can be safely infused into mice via tail-vein in an injection volume of 100 μl. Tumor growth or killing was quantitatively evaluated by whole body luminescence imaging, performed 1–2 times a week up to 8 weeks post tumor xenograft (Fig. 3A,D). Selected mice were also subjected to PET/CT imaging using ^18^F-NOTA(1,4,7-triazacyclononane-N,N′,N″-triacetic acid)-octreotide (NOTAOCT)^15,16^ to follow CAR T cell expansion and distribution (Fig. 3B,C). NOTAOCT was used instead of clinically approved ^68^Ga-DOTATATE for a lower cost and ease of synthesis, while both radiotracers had been shown to provide comparable specificity and sensitivity in mice^8,15,16^. The time to peak T cell density occurred ~3-weeks post xenograft (approximately, 2 weeks after T cell infusion) in most subjects, which corresponded to the timepoint just after peak tumor burden. T cells then followed a contraction phase after tumor elimination, indicated by a gradual decrease in NOTAOCT uptake in lungs. Compared to the cohorts with no treatment (No T group) or NT, the cohorts of AIC100 1X and MTD exhibited complete or near-complete tumor elimination lasting variable times before tumor relapse, which was observed in some subjects. The rate of tumor killing was slightly faster in mice receiving the MTD dose; similarly, mice receiving the MTD dose showed a longer lasting response than mice treated with the 1X dose. *In vivo* efficacy of CAR T cells manufactured with or without spinoculation (P2 vs. P2b) was found to be comparable. Survival was monitored up to 100 days post tumor xenograft (Fig. 3E). Median survival times for NT groups (42 days) were longer than No T (34 days), which was ascribed to often-occurring allogeneic killing by T cells (p < 0.01 by log-rank (Mantel-Cox) test). Median survival time of AIC100 1X (72 days) was significantly longer than NT at the MTD (p < 0.01). In comparison, more than 50% of subjects treated at the AIC100 MTD dose survived beyond 100 days post xenograft (>100 days), which was significantly longer than AIC100 1X and NT at MTD groups (p < 0.0001).

### Cross-reaction of AIC100 with mouse ICAM-1

**Figure 4:**
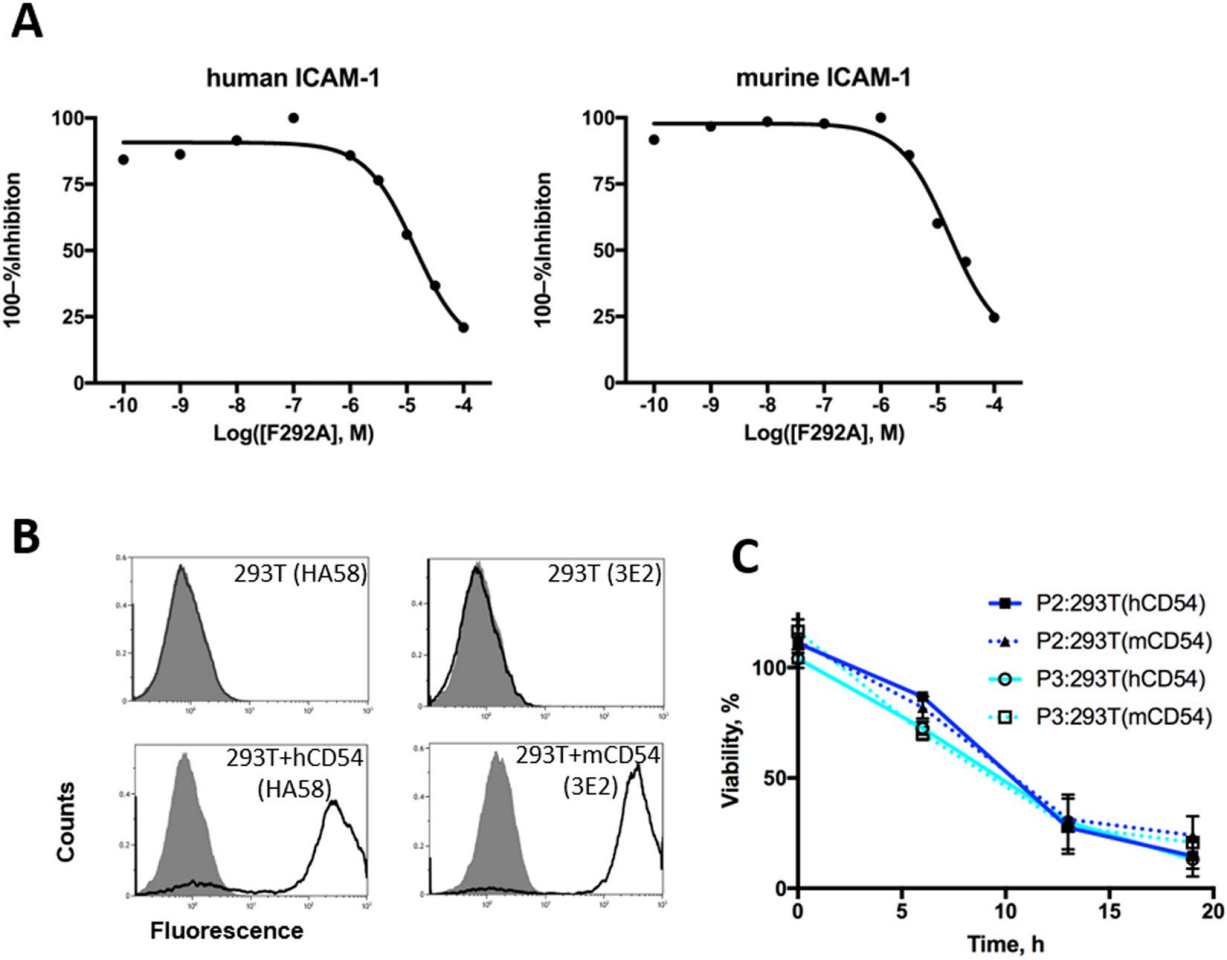
Comparable killing of target cells expressing human versus murine ICAM-1 by AIC100. (**A**) The affinity of soluble F292A to 293T cells stably transduced to express human and mouse ICAM-1 was determined by competition assay using flow cytometer. (**B**) Wild-type 293T cells and 293T cells transduced with human versus murine ICAM-1 were tested for ICAM-1 expression. Open and filled histograms correspond to unlabeled and anti-ICAM-1 antibody stained cells, respectively. Antibody clones used were HA58 (anti-human ICAM-1) and 3E2 (anti-murine ICAM-1). (**C**) E to T assay of AIC100 cells against ICAM-1 negative 293T, human ICAM-1 transduced 293T (hCD54), and murine ICAM-1 transduced 293T (mCD54). The ratio of T cells to target cells was 2.5:1.

We tested the ability of AIC100 to cross-react with mouse ICAM-1 in order to demonstrate that the toxicity of AIC100 from on-target, off-tumor killing can be examined in our preclinical model. This allows us to address concerns related to CAR T cells recognizing antigens expressed in non-tumor tissues by taking advantage of cross-reactivity between human and murine ICAM-1 by our CAR. The AIC100 CAR construct contains the I domain of integrin α_L_β_2_, which provides the sole molecular interaction with ICAM-1. I domains containing different mutations to increase affinity to ICAM-1 have been used to test binding to human and mouse ICAM-1. For example, I domains containing mutations such as F265S/F292G bound both human and mouse ICAM-1 at low-nanomolar affinities (Table 1; *K*_*D*_ = 6 nM vs. 2 nM, respectively)^17,18^. Another high affinity I domain induced by engineered disulfide bonds (K287C/K294C) bound comparably to human and mouse ICAM-1s (Table 1; 185 nM vs. 191 nM)^19^. Our CAR contains a much lower affinity I domain variant (F292A) that binds human ICAM-1 at micromolar affinity (20 μM by SPR)^18^. We also measured by flow cytometry (competition assay) the affinity of F292A to 293T cells transduced for expression of human and mouse ICAM-1. The affinity of the F292A I domain to human and mouse ICAM-1 was determined to be essentially identical (Table 1 & Fig. 4A; 12 vs 14 μM for human and mouse ICAM-1, respectively). We then examined if the comparable affinity of F292A I domain to human versus murine ICAM-1 led to comparable effector activity of CAR T cells against target cells. To eliminate any effect from inherent resistance of target cells to T cell cytotoxicity, we used 293T cells that were transduced with either human or mouse ICAM-1 (Fig. 4B). The rate of CAR T cell killing of 293T target cells transduced with either human or mouse ICAM-1 was indistinguishable (Fig. 4C).

**Table 1:** Affinity of LFA-1 I domain variants to human and mouse ICAM-1.

Name	Affinity
F292A to hCD54	20 μM (SPR)
F292A to hCD54	12 μM (Competition assay)
F292A to mCD54	14 μM (Competition assay)
K287C/K294C to hCD54	185 nM (SPR)
K287C/K294C to mCD54	191 nM (SPR)
F265S/F292G to hCD54	6 nM (SPR)
F265S/F292G to mCD54	2 nM (SPR)

### Comparable distribution of ICAM-1 in NSG mouse and human tissues

**Figure 5:**
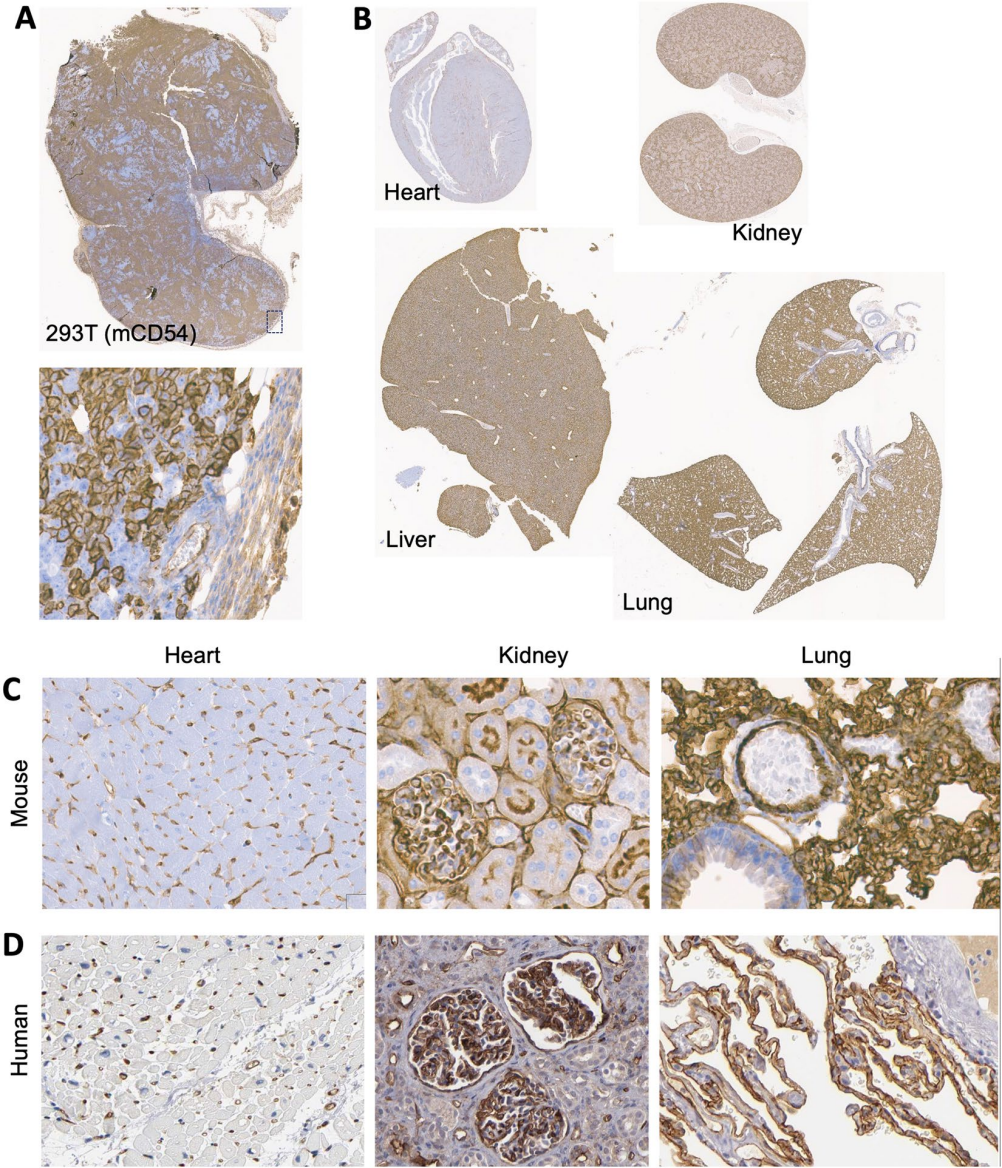
Comparison of the profile of ICAM-1 expression in tissues of human and mouse. (**A**) Specificity of anti-mouse ICAM-1 antibody (3E2) by immunostaining was first validated against subcutaneous tumors of 293T cells stably transduced with murine ICAM-1 (mCD54). ICAM-1 staining of the plasma membrane of 293T(mCD54) cells was clearly discerned from little to background staining of stroma cells within and at boundary of tumor. (**B**) Antibody staining of heart, liver, lungs, and kidneys was shown. (**C**) ICAM-1 staining was observed in glomeruli endothelial cells of the kidney and alveoli pneumocytes of the lung in mouse tissues. Heart tissue was devoid of specific ICAM-1staining. (**D**) Similar distribution of ICAM-1 expression in human tissues was observed. ICAM-1 staining of human tissues was obtained from Protein Atlas database (https://www.proteinatlas.org/ENSG00000090339-ICAM1/tissue).

After validating the comparable affinity and cytotoxicity by AIC100 toward target cells expressing human and mouse ICAM-1, we further demonstrated that the expression of ICAM-1 in NSG mice is similar to the known distribution of ICAM-1 in human tissues. Tissue expression of ICAM-1 in NSG mice was examined for lung and kidney, which were reported to have the highest normal expression of ICAM-1 in human tissues (https://www.proteinatlas.org/ENSG00000090339-ICAM1/tissue). As no standard immunohistochemistry antibody for murine ICAM-1 exists, we first validated the specificity of the antibody (clone 3E2) using subcutaneous tumor tissue of 293T cells expressing mouse ICAM-1. Plasma membrane staining of mouse ICAM-1 in 293T cells was confirmed to be specific with little to low background staining of fibroblast or other stroma cells (Fig. 5A). ICAM-1 expression in the lungs stained by the same antibody was similar to the staining pattern reported for human lungs, exhibiting darker staining of alveolar pneumocytes and weaker staining of other connective tissues (Fig. 5B–D). In mouse kidneys, ICAM-1 was distinct in glomerular endothelial cells and weaker in tubular epithelial cells, which is similar to the profile of ICAM-1 staining in human kidney.

### Toxicity of AIC100 against primary cells of human and mouse

**Figure 6:**
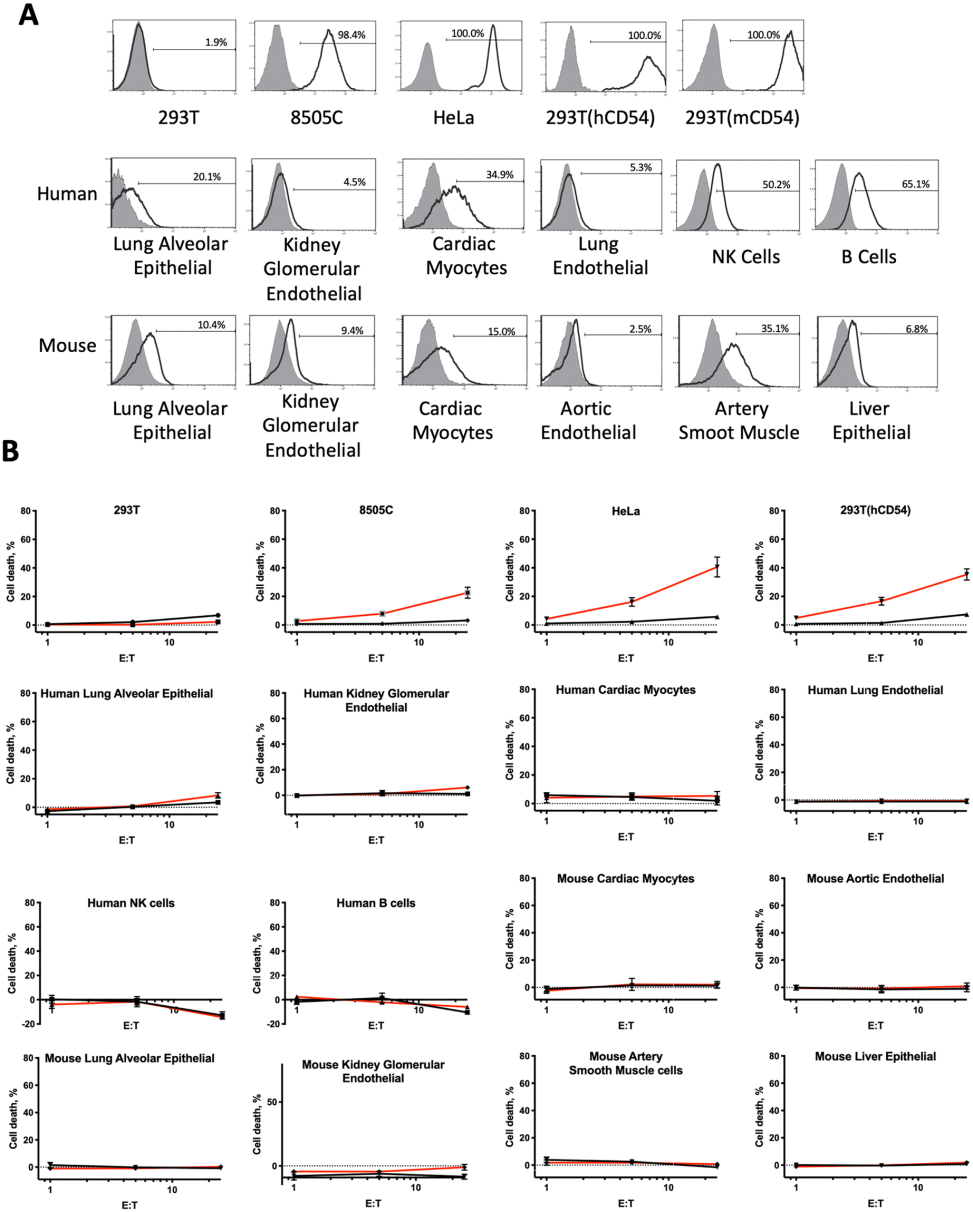
ICAM-1 expression and toxicity of AIC100 against primary cells of human and mouse tissues. (**A**) Primary human and mouse cells were tested for ICAM-1 expression by flow cytometry. Filled and open histograms correspond to unlabeled and anti-ICAM-1 antibody stained cells, respectively. Percent ICAM-1 positive is shown. (**B**) Target cells were incubated with T cells (red and black lines are of AIC100 and non-transduced T cells, respectively) for 4–5 h at 37 °C at the ratios of 25:1, 5:1, and 1:1. Cell death was calculated as %(treated − spontaneous)/(maximum − spontaneous), where treated, spontaneous, and maximum refer to the release of ^51^Cr from target cells with incubation with T cells, without T cells, and after lysis, respectively. Data are shown as mean ± sem.

To show selectivity of AIC100 toward cells with overexpressed ICAM-1 and its safety against normal cells, AIC100 cytotoxicity was examined with primary human and mouse cells derived from lung, liver, kidney, heart, and blood (Table 2 & Fig. 6). From our observation that the level of ICAM-1 increased with repeated passaging^5^, primary cells with not more than 5 passages were used for a viability assay. ICAM-1 expression of each type of cells was determined by flow cytometry using antibodies that were validated for specificity against 293T cells expressing human versus murine ICAM-1 (monoclonal antibodies HA58 and 3E2, as shown in Fig. 4B). ICAM-1 expression was detectable at low levels in most human cell types except endothelial cells from lung, and was similarly detectable in mouse cells (Fig. 6A). Overall, ICAM-1 expression in primary cells was lower than the levels measured in 8505C and HeLa. Cell viability was assessed by the amount of ^51^Chromium (^51^Cr) released into the media as a readout for cell death. (This is in contrast to the luminescence assay (e.g., data shown in Fig. 2) that measures the percent of viable cells.) Similar to the luminescence-based E to T assays, ^51^Cr release from 293T, 8505C, and HeLa cell lines after incubation with AIC100 and NT T cells at 25:1, 5:1, and 1:1 ratios was specific to target cells with higher ICAM-1 expression; *i.e*., higher levels of ^51^Cr release were observed with target cells expressing higher levels of ICAM-1 as well as in cultures with a higher E to T ratio (Fig. 6B & Table 2). In comparison, the level of cell death was much lower in normal human primary cells (less than 8.5%) and non-detectable in normal murine primary cells (less than 2%). No significant difference in cell death induced by AIC100 versus NT was found in either human or murine primary cells, although several human primary cells seemed to be slightly more susceptible to killing by AIC100. We suspect that higher cell death of human cells by AIC100 is partly due to allogeneic TCR-mediated killing, which can be boosted by T cell stimulatory signals of the CAR; this phenomenon was also apparent against ICAM-1 negative 293T cells measured over 24 h (Fig. 2). We also noted that the level of ^51^Cr release from NK and B cells incubated with T cells at 25:1 was even lower compared to target cell only conditions. We speculate that this was likely due to the uptake of spontaneously released ^51^Cr from NK and B cells by more abundant T cells, resulting in lower amounts of ^51^Cr in media than those from 5:1 and 1:1 conditions.

**Table 2:** AIC100 toxicity on cell lines and primary cells of human and mouse tissues.

Target cells	%cell death by NT*	%cell death by CAR T*	significance by t-test**
Cell lines			
293T	3.3 ± 1.3 (10)	2.2 ± 0.6 (8)	n.s.
293T(hCD54)	7.4 ± 0.9 (8)	35.4 ± 4.0 (10)	<0.0001
8505C	3.2 ± 1.2 (8)	22.6 ± 3.8 (10)	0.0005
HeLa	5.7 ± 1.1 (9)	40.6 ± 6.9 (10)	0.0002
**Human primary cells**			
Lung Microvascular Endothelial Cells	−1.2 ± 1.5 (6)	−0.7 ± 1.4 (8)	n.s.
Kidney Glomerular Endothelial Cells	1.1 ± 0.7 (4)	6.1 ± 1.5 (7)	n.s.
Lung Alveolar Epithelial Cells	3.4 ± 1.4 (7)	8.4 ± 1.9 (10)	n.s.
Cardiac Myocytes	1.9 ± 3.0 (4)	5.4 ± 3.0 (5)	n.s.
NK cells	−12.9 ± 2.9 (4)	−14.5 ± 1.7 (5)	n.s.
B cells	−10.4 ± 0.3 (4)	−5.9 ± 1.9 (5)	n.s.
**Mouse (BALB/c) primary cells**			
Aortic Endothelial Cells	−1.0 ± 2.2 (4)	0.9 ± 2.3 (5)	n.s.
Kidney Glomerular Endothelial Cells	−8.4 ± 1.9 (4)	−1.0 ± 2.2 (5)	n.s.
Lung Alveolar Epithelial Cells	−0.8 ± 0.9 (6)	0.3 ± 0.8 (8)	n.s.
Artery Smooth Muscle Cells	−1.5 ± 1.4 (4)	0.7 ± 1.7 (4)	n.s.
Liver Epithelial Cells	0.9 ± 0.3 (4)	1.8 ± 1.1 (6)	n.s.
Cardiac Myocytes	1.3 ± 1.9 (4)	2.0 ± 2.4 (4)	n.s.

*Percent cell death is shown as mean ± sem (sample size). Negative values indicate that spontaneous release of ^51^Cr from target with T cells is lower than from target only condition. **Student t-test (unpaired, two-tailed) to examine significance (p < 0.05) of cell death by non-transduced (NT) T cells versus CAR T cells at E to T ratio of 25:1. n.s. = not significant. P values are shown for significance.

### Impact of dual expression of SSTR2 with CAR in AIC100

**Figure 7:**
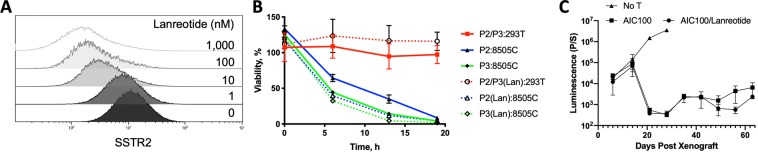
Effect of SSTR2 expression and activation on CAR T cell activity *in vitro* and *in vivo*. (**A**) The level of antibody binding to SSTR2 was measured to assess SSTR2 internalization induced by SSTR2 agonist (lanreotide). (**B**) E to T assay of AIC100 cells with or without 1 μM of lanreotide against 293T and 8505C cells. The ratio of T cells to target cells was 2.5:1 (n = 3 each for P2 and P3). (**C**) A weekly measurement of whole body luminescence to detect tumor growth or killing by AIC100 (P2 and P2b) at MTD doses with or without lanreotide. Lanreotide was administered to mice by intraperitoneal injection at days of 9, 19, 23, 34, and 40 post tumor xenograft. n = 5 for AIC100/Lanreotide; n = 3 for AIC100; n = 4 for No T. Significant difference (p = 0.01) for No T vs. AIC100 or AIC100/Lanreotide; no significance for AIC100 vs. AIC100/Lanreotide by one-way ANOVA with Tukey’s multiple comparisons test.

SSTR2 is coexpressed with the CAR in AIC100 cells, which was introduced to track T cells after infusion by PET/CT imaging using the SSTR2-specific radiotracer ^68^Ga-DOTATATE (approved by FDA in 2018 for imaging SSTR2 positive neuroendocrine cancer^9,20^) or other specific radiotracer analogues such as ^68^Ga-DOTATOC or NOTAOCT^8,10^. In order to determine if SSTR2 activation would negatively impact the function of CAR T cells, we examined the efficacy of AIC100 *in vitro* and *in vivo* with the addition of the SSTR2 agonist Lanreotide (Sigma). Similar to our previous study demonstrating octreotide-induced internalization of SSTR2 in Jurkat T cells^10^, lanreotide caused dose-dependent internalization of SSTR2 in CAR T cells, indicated by gradual reduction of antibody binding to SSTR2 (Fig. 7A). Also in agreement with our previous study^10^, the addition of SSTR2 agonist (lanreotide at 1 μM) did not alter the rate of AIC100 killing of 8505C target cells (Fig. 7B). Furthermore, a weekly intraperitoneal injection of lanreotide (10 μl at 50 mg/ml) did not change the rate of tumor elimination by AIC100 *in vivo* (Fig. 7C). The lack of effect of SSTR2 expression or activation by a SSTR2 agonist on AIC100 activity was consistent with the observation that there was no appreciable difference in tumor killing between cohorts subjected to PET/CT imaging using SSTR2 tracer agonists (e.g., ^68^Ga-DOTATOC or NOTAOCT) versus those that did not receive the tracers in our previous^8,10^ and current studies.

### Toxicology study

**Figure 8:**
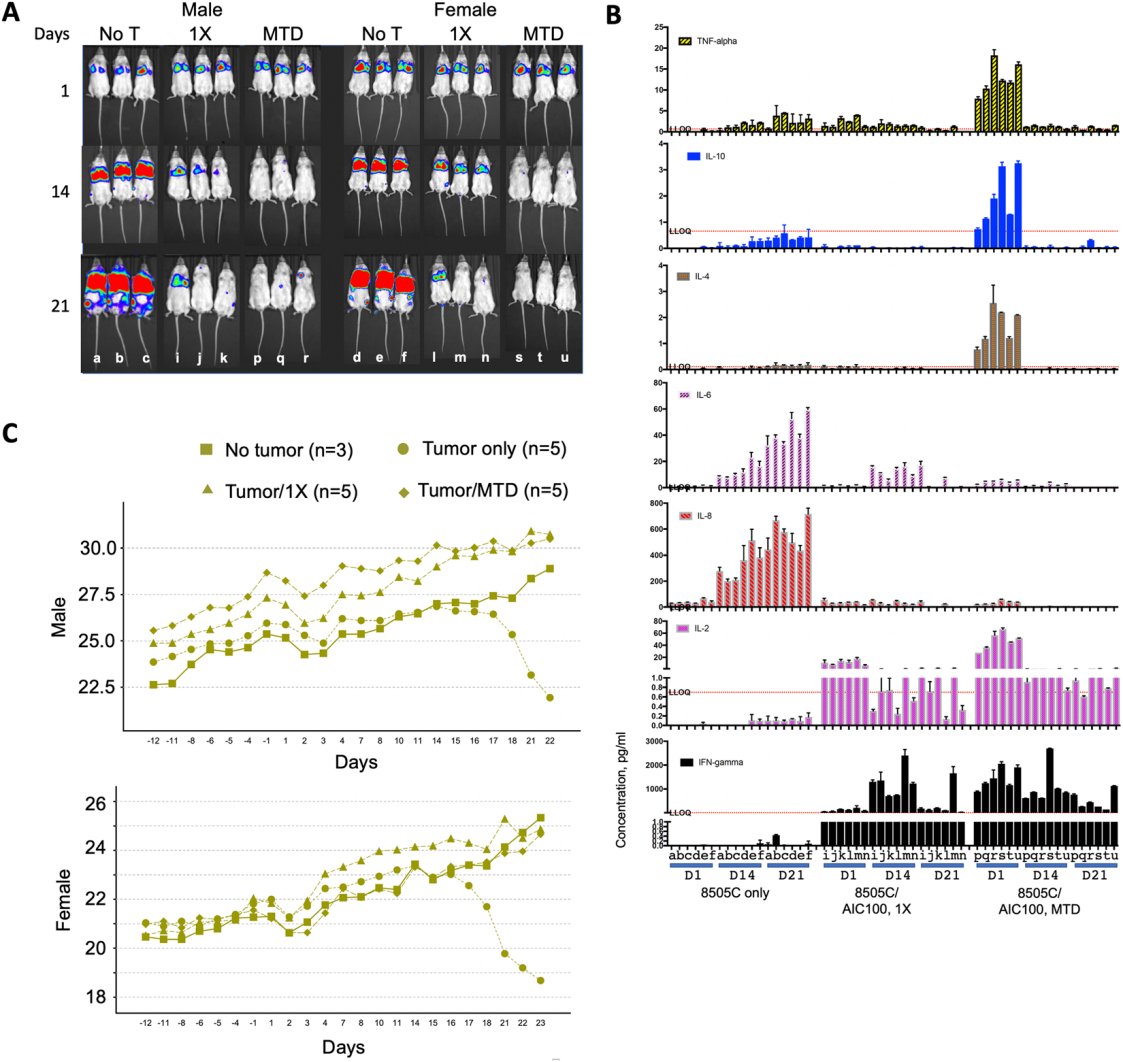
GLP toxicology study to evaluate AIC100 efficacy and safety in male and female mice. (**A**) A change in 8505C tumor burden was measured by whole body luminescence imaging of tumor growth. Days indicate time elapsed after infusion of AIC100. 1X and MTD doses are identical to previously defined. Animals were labeled as ‘a’, ‘b’, ‘c’, etc. to match luminescence with cytokine levels. (**B**) Cytokines were measured from the peripheral blood collected from the animals subjected to whole body imaging. Each sample was measured twice. LLOQ = lower limit of quantification. (**C**) The body weight was recorded for the cohorts of mice with no tumor, mice with tumor only, mice with tumor and treated with 1X AIC100, and mice with tumor and treated with AIC100 at MTD.

The toxicity study of AIC100 in mice was conducted to support an Investigational New Drug (IND) application for the treatment of advanced thyroid cancer in complete accordance with FDA Good Laboratory Practice (GLP) regulations. This study was designed to evaluate the acute (24 h) and delayed (2 and 3 weeks) toxicity of a single intravenous injection of AIC100 cells at 1X dose and MTD in male and female NSG mice (n = 78 each) implanted with 8505C cancer cells (Fig. 8). The full report from this study will be included in our IND application; herein, we show AIC100 tumor killing assessed by whole body imaging, a change in body weight, and cytokine profiles. Similar to the kinetics of tumor killing observed under non-GLP conditions (Fig. 3A), a 1X dose of AIC100 in both male and female mice led to significant tumor reduction at 2 weeks after T cell infusion and almost complete tumor elimination at 3 weeks in 2 out of 3 subjects that were scanned (Fig. 8A). The rate of tumor killing was more rapid in mice treated with AIC100 at the MTD dose, and by 3 weeks near complete tumor elimination was observed in all subjects. To examine a correlation between AIC100 efficacy and toxicity with cytokine profiles, blood was taken from mice that were subjected to whole body imaging and analyzed for different cytokines (Fig. 8B). IFN-γ levels in blood from mice treated with 1X AIC100 peaked at 2 weeks post infusion and subsequently decreased to below 100 pg/ml for 5 out of 6 subjects. Compared to the 1X cohort, IFN-γ levels in mice treated with the MTD dose displayed the highest levels 1 day after T cell infusion, which then gradually declined to below 1 ng/ml for 5 out of 6 subjects. IL-2 levels in both groups were much lower and gradually decreased from day 1 levels. IL-8 and IL-6, which we confirmed to be secreted by 8505C tumor cells (~20 ng/ml/24 h for IL-8 and ~2 ng/ml/24 h for IL-6 in tissue culture), closely reflected the change in tumor burden. IL-10, IL-4, and TNF-α were measured above the detection limit mainly for the MTD cohort 1 day after CAR T infusion, which reduced to lower limit of detection levels at 14 and 21 days post CAR T infusion. Three other cytokines (IL-1β, IL-12p70, IL-13) in 1X and MTD cohorts were at lower limit of detection levels. Contrary to ~20% loss of body weight in the tumor only cohort, the AIC100 cohorts at both doses displayed a gradual increase in body weight that was indistinguishable from the control cohort with no tumor (Fig. 8C).

## Discussion

CAR T cells are being developed as a new treatment modality for many different types of cancers that are refractory to existing treatments. One of the biggest challenges in CAR T cell therapy is ensuring safety, as T cells engineered to recognize antigens expressed in normal tissues can potentially be fatal to patients. The process of CAR T manufacturing is also very complex, as T cells need to be modified, expanded, and formulated to be stable and functional upon infusion into patients. Encouraged by robust killing of one of the most malignant tumors, *i.e*., anaplastic thyroid cancer^5^, we developed a clinically compliant protocol for CAR T manufacturing for our planned clinical study in patients with any stage of anaplastic thyroid cancer, or refractory or relapsed aggressive thyroid cancers. The automated process of CAR T manufacturing was highly reproducible and robust in terms of fold-expansion, viability, and the level of CAR expression. The final product was then cryopreserved in formulation solution to be used directly for infusion into patients after thawing.

CAR T cells manufactured via a GMP-compliant process were then tested for pharmacology and toxicology studies. For *in vivo* studies, two different CAR T cell doses were used - one representing a dose for producing significant tumor killing and the other dose that would be the maximum number of cells that can be safely injected into mice. The lower dose of CAR T cells produced substantial to complete eradication of tumors, although tumor relapse was more frequent than in MTD cohorts. Treatment at the higher dose led to more rapid and complete eradication of tumors, which resulted in a longer remission and a significantly higher survival benefit. Incorporation of SSTR2 into the lentiviral vector demonstrated the value of T cell imaging for prediction of tumor response and possibly toxicity indicated by off-tumor expansion of CAR T cells. Similar to the previous finding of biphasic T cell kinetics^8,10^, T cell expansion and contraction were clearly observed in subjects with tumor elimination. The use of SSTR2 can be extended beyond imaging for other purposes such as suicide switch. Similar to the use of monoclonal antibody (cetuximab) against truncated epidermal growth factor receptor as a suicide strategy^21^, SSTR2 antibodies that possess antibody-dependent cell-mediated cytotoxicity may be developed and used for eliminating SSTR2-expressing CAR T cells. With the FDA approval of ^177^Lutetium-DOTATATE to treat SSTR2 positive neuroendocrine tumor^22^, one may explore an optimum dose of this drug to eliminate SSTR2-expressing CAR T cells with least side effects.

CAR T cells manufactured from seven different runs using leukopaks collected from five unique donors produced fairly uniform efficacy in terms of rate of tumor killing, suppression of tumor relapse, and survival. This functional consistency could be attributed to the similarity in the level of CAR expression, cell viability, and CD8^+^ to CD4^+^ T cell ratios. Furthermore, CAR T cells manufactured through the current automated process involving longer expansion times (~10 days) and cryopreservation were found to be comparably efficacious to CAR T cells that were manufactured in standard tissue-culture flasks for a shorter expansion period (less than 7 days) and used without undergoing cryopreservation. In the current protocol, ~30 million T cells were used for initial seeding, which was expanded to ~3 billion T cells, corresponding to 100-fold expansion in cell number. With more evidence for higher efficacy for CAR T cells with shorter days of manufacturing, our protocol is readily amenable for ~5-day manufacturing schedule by increasing a starting number of cells to ~100 million and harvest products when the cell number reaches roughly 1 billion T cells (10-fold expansion).

Heterogeneity of tumor antigen expression, which can be further driven by antigen-specific treatment, is a significant problem for all CAR T-based cancer therapy (including CD19 CAR T^23^). The distribution of ICAM-1 and its role in physiology and disease processes have been extensively studied using *in vitro* and *in vivo* models; specific examples include the process of leukocyte adhesion to inflamed endothelium mediated by interaction between LFA-1 and ICAM-1^24^, induction of ICAM-1 in inflamed tissues and cells^25^ and ICAM-1 specific drug delivery^26–29^. As many studies report the association between tumor relapse or drug resistance and ICAM-1 overexpression, ICAM-1 targeting has obvious advantages even if ICAM-1 is not uniformly elevated in early stage cancers. In the course of CAR T killing of tumor cells, low ICAM-1 tumors within or near high ICAM-1 tumors may become susceptible to CAR T cell killing due to induction of ICAM-1 by T cell cytokines such as IFN-γ. However, ICAM-1 is also expressed in immune, endothelial, and some epithelial cells, and is upregulated by diverse inflammatory conditions^30,31^. Despite the concerns of targeting a molecule that is expressed in healthy cells, previous clinical studies have shown that monoclonal antibodies targeting ICAM-1 were generally well tolerated in various clinical settings. For example, an anti-ICAM-1 monoclonal antibody (R6.5, enlimomab**)** was tested in clinical trials in patients with rheumatoid arthritis, acute stroke, and to treat acute rejection of renal transplants and burn injury^32–35^. Enlimomab, which elicits complement-mediated neutrophil activation, was well tolerated and led to clinical benefits in arthritis, stroke, and burn recovery. More recently, an antibody against ICAM-1 was developed for its cytotoxic activity against multiple myeloma cells, which appeared to be mediated by antibody-dependent cell cytotoxicity^36,37^. In phase I and II trials in patients with relapsed/refractory multiple myeloma and smoldering myeloma, this antibody (BI-505) was also well tolerated at the highest dose of 20 mg/kg with mild to moderate, mostly infusion-related adverse events. The safety profiles of antibody-dependent cell cytotoxicity or complement dependent cytotoxicity-inducing antibodies against ICAM-1 even at doses that are likely to saturate all ICAM-1 molecules in the human body support the idea that ICAM-1 may be safely targeted in settings where ICAM-1 is overexpressed and dysregulated.

Given the cross-reactivity of the human LFA-1 I domain with murine ICAM-1 and comparable CAR T cytotoxicity towards target cells expressing either human or murine ICAM-1, we were able to show the utility of our mouse model to examine the on-target, off-tumor toxicity of our CAR T cells concurrently with on-target, on-tumor efficacy. The finding that affinity-tuned CAR T cells induced minimum killing of human primary cells (e.g., alveolar epithelial and glomeruli endothelial cells) that express ICAM-1 at the highest levels relative to other normal cells supports the idea that ICAM-1 expression in normal cells falls below the threshold to induce full activation of micromolar affinity ICAM-1 CAR T cells. This study provides a strategy to develop CAR T cells against antigens that are over-expressed in tumors yet also found at lower levels in normal cells and tissues. Although affinity-tuning of CARs to avoid normal tissues has been demonstrated previously^38,39^, using a CAR that cross-reacts with mouse antigens as in the current study more stringently tests how CAR affinity affects both efficacy and safety. This was illustrated from our previous study by the finding that while low nanomolar affinity CAR T cells uniformly resulted in fatality, micromolar affinity CAR T cells produced effective tumor elimination in the absence of toxicity^8^. In summary, we show that micromolar affinity CARs limit CAR T cell killing to targets cells expressing antigens above a threshold antigen density. Given the paucity of antigens that are exclusively expressed on tumor cells, our strategy may be advantageous in the design of many CARs targeting solid tumors.

## Materials and Methods

### Mammalian cell culture

Parental HeLa (ATCC), HEK 293T (ATCC), and 8505C (DSMZ) cells were transduced with lentivirus encoding Firefly Luciferase-F2A-GFP (Biosetta) and sorted by fluorescence activated cell sorting (FACS) to select GFP expressing cells. HeLa and 293T cells were cultured in Advanced Dulbecco’s Modified Eagle Medium containing 10% (v/v) fetal bovine serum (FBS), 2 mM L-alanyl-L-glutamine dipeptide (Gibco), and 100 U/ml Penicillin-Streptomycin (Pen/Strep) (Gibco). 8505C-FLuc^+^GFP^+^ cells were cultured in RPMI-1640 supplemented with 10% (v/v) FBS, 2 mM L-alanyl-L-glutamine dipeptide, and 100 U/ml Pen/Strep. Human primary cells include: lung microvascular endothelial cells (H-6011, Cell Biologics, Inc), lung alveolar epithelial cells (H-6053, Cell Biologics, Inc), kidney glomerular endothelial cells (H-6014G, Cell Biologics, Inc) and cardiac myocytes (C-12810, PromoCell GmbH). Mouse (BALB/c) primary cells include: aortic endothelial cells (BALB-5052, Cell Biologics, Inc), kidney glomerular endothelial cells (BALB-5014G), lung alveolar epithelial cells (BALB-5053), artery smooth muscle cells (BALB-5081), liver epithelial cells (BALB-5044), and cardiac myocytes (BALB-5228). Primary cells were cultured adhered to 0.1% gelatin pre-coated dishes in media recommended for each cell type according to the suppliers. All cells were incubated at 37 °C in a 5% CO_2_ humidified incubator. Leukopak was obtained commercially (Bio Specialty Corporation, PA), which was delivered to the manufacturing site within 4 hours of blood collection. CD4/CD8 sorted leukopak cells were cultured in TexMACS medium (Miltenyi) supplemented with 3% human AB serum (Sigma or Valley Biomedical) and 12.5 ng/mL IL-7, 12.5 ng/mL IL-15 (Miltenyi).

### Lentiviral vector and manufacturing of CAR T cells byprodigy

Lentivirus suitable for IND enabling studies was manufactured using a third generation lentiviral design (Lentigen Technology, Inc., a subsidiary of Miltenyi Biotec GmbH (Miltenyi)). The titer of the virus was determined by qPCR on 293 cells. Fresh leukopak was prepared for cryopreservation in cryobags (CryoMACS 250) by diluting prechilled 20 ml of the leukopak with 40 ml of Cryostor CS10 (BioLife Solutions), preserving cells at 6.7% (v/v) DMSO. The bag was frozen at −80 °C overnight and then transferred to a liquid nitrogen tank for a long-term storage. Thawed, diluted leukopak cells were loaded into the T Cell Transduction Process v2.0 (TCT v2.0) on Prodigy. A single TS520 tubing set (Miltenyi) was used for the duration of the process, beginning with immunomagnetic enrichment of CD4^+^ and CD8^+^ cells. 30 × 10^6^ viable cells were seeded in the Prodigy Culture Chamber at the end of Day 0 post-enrichment and activated using MACS GMP TransAct reagent (Miltenyi). On Day 1 and Day 2, transduction of T cells was performed using 1.5 ml lentivirus vector each day (total 3 ml) in 10 ml of medium or PlasmaLyte A (Baxter)/1% human serum albumin (HSA, Gemini Bio-Products). (For spinoculation transduction, a spin step was added to the protocol upon lentivirus addition.) Cells were expanded in TexMACS medium supplemented with 3% human AB serum and 12.5 ng/mL IL-7, 12.5 ng/mL IL-15 until harvest on Day ~10. Final products were cryopreserved in a 1:2 mixture of PlasmaLyte A (+1% HSA) and CS10 at indicated doses.

### E:T assay with cell lines

HeLa-FLuc^+^GFP^+^, 8505c-FLuc^+^GFP^+^ or HEK 293T-FLuc^+^GFP^+^ cells were co-cultured with either non-transduced or CAR T cells at the indicated E:T ratios. Prodigy-manufactured CAR T cells were freshly thawed and cultured for >24 h in TexMACS/IL-7/IL-15 media before being added to target cells. Co-cultures were carried out in TexMACS media with 5% human serum albumin with 150 µg/ml D-Luciferin (Gold Biotechnology) and no cytokine supplementation. Luminescence was measured using a plate reader (TECAN infinite M1000 PRO) with readings in each E:T condition normalized to the target only controls.

### Chromium-51 (^51^Cr) release assay

CAR T cytotoxicity against primary cells derived from human and mouse tissues was assessed by *in vitro* ^51^Cr (PerkinElmer) release assay. Human NK and B cells were purified from leukopak using B and NK cell purification kits (Cat no. 130-092-657 and 130-091-151, respectfully, Miltenyi). Target cells were labeled in suspension with 50 µCi of ^51^Cr for 2 h at 37 °C and washed three times before use. Effector cells were incubated with corresponding targets at the indicated E:T ratios for 4 h at 37 °C. Samples were centrifuged and 50 µl of supernatant harvested. ^51^Cr release into media was measured by gamma counter (Walac Wizard 3.0 gamma counter). Specific lysis was calculated using the equation: 100 × [(experimental release − spontaneous release)/(maximum release − spontaneous release)], where experimental release represents ^51^Cr release from the target cells co-incubated with T cells, spontaneous release is from target cells without T cells, and maximum release is from target cells after incubation with 10% lysis buffer (Triton X-100 final concentration of 2%).

### 8505C mouse model, whole-body luminescence measurement of tumor growth

NSG mice were injected via tail vein with 0.5 × 10^6^ 8505c-FLuc^+^GFP^+^ cells. 5 days after tumor cell injection, cryopreserved AIC100 was quickly thawed by hand and used for injection via tail vein. Tumor growth or killing in live mice was assessed by luminescence imaging using a whole body optical imager (*In-Vivo* Extreme 4MP, Bruker). During luminescence scan, mice were first anesthetized with 3% isoflurane at 2 L/min O_2_ and subsequently maintained at 2% isoflurane at 2 L/min O_2._ 3 mg of D-luciferin (GoldBio) in 200 μl was injected intraperitoneally, and luminescence over the lungs or the entire mouse body was used to estimate tumor burden.

### Labeling of ^18^F-NOTA-octreotide (NOTAOCT)

NOTAOCT (1,4,7-Triazacyclononane-1,4,7-triacetic acid-octreotide^15,40^, GMP grade) was obtained as a 1 mg lyophilized powder (cat #9762, ABX Pharmaceuticals). NOTAOCT was chelated and purified with Fluorine-18 (^18^F) following the steps described previously^8,41^. The purity of NOTAOCT was validated by reverse phase HPLC.

### PET/CT imaging

PET/CT images were acquired using a micro-PET/CT scanner (Inveon, Siemens) at 1–2 h post NOTAOCT injection following a procedure described previously^10^. A reference was included using a tube containing 100 µl of 10% ID/cm^3^ for quantification of NOTAOCT uptake *in vivo*. To compute radiotracer uptake by systemic 8505C tumor models, the ellipsoidal regions of interests were drawn (Amide) separately on the left and right sides of the lungs to enclose most of the five lobes of the mouse lungs. Visualization and analyses of PET/CT images were performed using Amide.

### Measurement of affinity of F292A I domain to ICAM-1

Human LFA-1 I domains (F265S and F292A) were produced as bacterial inclusion body and refolded as described previously^18^. To measure I domain binding to cells, HEK 293T cells were transduced by lentivirus to express full-length ICAM-1 of human and mouse sequences. ICAM-1 expressing cells were then sorted by magnetic beads to enrich ICAM-1 positive cells. I domains were labeled with Alexa Fluor 488 dye (ThermoFisher) to avoid using secondary labels. The equilibrium dissociation constant (*K*_D_) of F265S was measured by a saturation binding assay using flow cytometry. 1 × 10^4^ 293T-hCD54, 293T-mCD54, or 293T cells were stained with 2-fold serially diluted Alexa Fluor 488 labeled F265S (2 µM to 0.125 µM). After 15 min incubation, cells were washed and analyzed by flow cytometer. *K*_D_ values of F265S to hCD54 and mCD54 were estimated by analysis of median fluorescence intensities (MFI) for each sample set with Prism 7 (GraphPad). In a competition binding assay, 1 × 10^4^ 293T-hCD54 and 293T-mCD54 cells were incubated for 15 min in 50 nM Alexa Fluor 488 labeled F265S and serially diluted F292A (10^−4^ to 10^−10^ M). After washing, cell samples were analyzed by flow cytometer. The half-maximal inhibitory concentrations (IC_50_) were determined using Prism 7. The affinity of F292A was calculated using equation: *K*_i_ = IC_50_/(1 + [L]/*K*_D_), where *K*_D_ represents the affinity of F265S to I domain and [L] represents Alexa Fluor 488 labeled F265S concentration (50 nM).

### Flow cytometry

Antibodies against human ICAM-1 (HA58, Biolegend) and mouse ICAM-1 (3E2, BD Biosciences) were used for confirming ICAM-1 expression in transduced 293T cells. T cells were labeled with antibodies against CD3, CD4, and CD8 (Biolegend). CAR expression was confirmed by antibodies against Myc (Cat #: 130-092-472, Miltenyi) and SSTR2 (FAB4224A, R&D Systems). Cell viability was measured by live staining with 1 µg/ml Propidium Iodide (Invitrogen, P3566). Flow cytometry gates were first determined by live cell gating (Propidium Iodide negative) and subsequently by staining of respective antibodies.

### Vector copy number (VCN)

VCN assay was performed using droplet digital PCR (ddPCR) (Bio-Rad) technique using primers specific to PTBP2 (Polypyrimidine Tract Binding Protein 2, reference gene; forward 5′-TCTCCATTCCCTATGTTCATGC, reverse 5′-GTTCCCGCAGAATGGTGAGGTG) and WPRE (viral gene; forward 5′-CCGTTGTCAGGCAACGTG, reverse 5′-AGCTGACAGGTGGTGGCAAT). Genomic DNA was isolated from 10^6^ cells (Qiagen), suspended in 100–200 μl water and serially diluted to get 10^4^-10^2^ copy number per reaction. VCN was calculated as the ratio of WPRE copy number to PTBP2 copy number multiplied by a factor ‘2’. The average value was obtained from triplicate or three serially diluted samples.

### Histology

Mouse organs were harvested to examine ICAM-1 expression. After euthanasia, mouse lungs were perfused via trachea with 4% paraformaldehyde, and each of the five lobes was separated post fixation and embedded in paraffin. Other tissues (kidneys, liver, heart) were harvested and processed for histology (Molecular Cytology Core Facility, MSK). Histological analysis was performed by an experienced pathologist.

### GLP toxicology study

The study to evaluate the acute and delayed toxicity of a single intravenous infusion of AIC100 in NSG mice was performed by the Antitumor Assessment Core (ATAC) Facility at Memorial Sloan Kettering Cancer Center under GLP conditions. Human cytokines in mouse plasma samples were measured by multiplex immunoassay (MSD V-Plex) using human proinflammatory panel 1 (IFN-γ, IL-1β, IL-2, IL-4, IL-6, IL-8, IL-10, IL-12p70, IL-13, TNF-α).

### Statistical analysis

Student’s t-test, ANOVA, and survival analysis were performed using Prism 7 on data indicated. A ‘p’ value less than 0.05 was considered significant.

### Study approval

All animal experiments were performed in accordance with the National Institute of Health’s Guide for the Care and Use of Laboratory Animals. Animal handling protocols were approved by the Institutional Laboratory Animal Use and Care Committee of Weill Cornell Medicine (Permit Number: 2012-0063).

## Data Availability

All data generated or analyzed during this study are included in the published article.
