# Publisher Correction: Manufacturing and preclinical validation of CAR T cells targeting ICAM-1 for advanced thyroid cancer therapy

**DOI:** 10.1038/s41598-020-69586-8

**Published:** 2020-07-27

**Authors:** Yogindra Vedvyas, Jaclyn E. McCloskey, Yanping Yang, Irene M. Min, Thomas J. Fahey, Rasa Zarnegar, Yen-Michael S. Hsu, Jing-Mei Hsu, Koen van Basien, Ian Gaudet, Ping Law, Nak Joon Kim, Eric von Hofe, Moonsoo M. Jin

**Affiliations:** 1 Department of Radiology, Molecular Imaging Innovations Institute, New York, NY 10065 USA; 2 Department of Surgery, New York, NY 10065 USA; 3 Department of Pathology and Laboratory Medicine, New York, NY 10065 USA; 4 Department of Hematology/Oncology, Weill Cornell Medicine, New York, NY 10065 USA; 5 Miltenyi Biotec Inc., Sunnyvale, CA 94089 USA; 6 AffyImmune Therapeutics, Inc., Natick, MA 01760 USA

Correction to: *Scientific Reports* https://doi.org/10.1038/s41598-019-46938-7, published online 23 July 2020.

In the original version of this Article, the author Koen van Besien was incorrectly indexed. This error has now been corrected.

